# Single-center experience of voclosporin use for pediatric lupus nephritis: a case series

**DOI:** 10.1093/rheumatology/keag125

**Published:** 2026-03-15

**Authors:** Ran Hazan, Nazlican Tang Civilibal, Kevin Baszis, Tarin M Bigley

**Affiliations:** Division of Rheumatology and Immunology, Department of Pediatrics, Washington University School of Medicine, St. Louis, MO, United States; Division of Rheumatology and Immunology, Department of Pediatrics, Washington University School of Medicine, St. Louis, MO, United States; Division of Rheumatology and Immunology, Department of Pediatrics, Washington University School of Medicine, St. Louis, MO, United States; Division of Rheumatology and Immunology, Department of Pediatrics, Washington University School of Medicine, St. Louis, MO, United States; Department of Pathology and Immunology, Washington University School of Medicine, St. Louis, MO, United States; Department of Molecular Microbiology, Washington University School of Medicine, St. Louis, MO, United States

Rheumatology key message

- Voclosporin, a calcineurin inhibitor, is a safe and effective treatment option for paediatric patients with lupus nephritis.

Dear Editor, Systemic lupus erythematosus (SLE) is a heterogeneous disease characterized by multi-organ autoimmunity. Paediatric lupus nephritis (LN) represents a particularly severe manifestation of SLE in children, characterized by a more aggressive disease course compared with adult-onset LN. Approximately 50–60% of children with SLE develop LN, significantly contributing to morbidity and mortality in this population [1, 2]. The management of paediatric LN involves induction therapy, typically corticosteroids combined with either mycophenolate mofetil or intravenous cyclophosphamide [3]. Maintenance therapy often involves the addition of hydroxychloroquine as well as lower doses of immunosuppressive agents to prevent disease flares and minimize long-term organ damage. Recent guidelines recommend the addition of a calcineurin inhibitor as part of the management of LN in adults [4].

Voclosporin is a calcineurin inhibitor that has demonstrated efficacy in treating LN. Voclosporin, approved by the FDA in January 2021 for adult patients with LN, has shown promise in clinical trials for its ability to improve renal response rates when added to standard therapy with MMF and glucocorticoids. The AURA-LV and AURORA 1 trial demonstrated that voclosporin significantly increased complete renal response rates compared with placebo, with a comparable safety profile [5, 6]. The KDIGO 2024 Clinical Practice Guideline for the Management of LN supports the use of voclosporin in combination with MMF and glucocorticoids for adult patients with active LN [4]. Voclosporin has a favourable pharmacokinetic profile and does not require therapeutic drug monitoring, making it an attractive option for use in paediatrics. Although paediatric patients were underrepresented in the pivotal trials, the drug’s efficacy and safety in adults suggest potential benefits for younger patients, warranting further investigation and potential off-label use [7]. A phase III clinical trial (NCT05288855) to evaluate the efficacy and safety of voclosporin in patients aged 12–18 is currently underway. We report five clinical cases of paediatric patients diagnosed with LN who underwent successful treatment using voclosporin (Fig. 1). The patients had varying manifestations of SLE, variable kidney pathology class and chronicity, and variations in therapeutic approaches before voclosporin (Supplementary Table S1, Supplementary Fig. S1).

**Figure 1:**
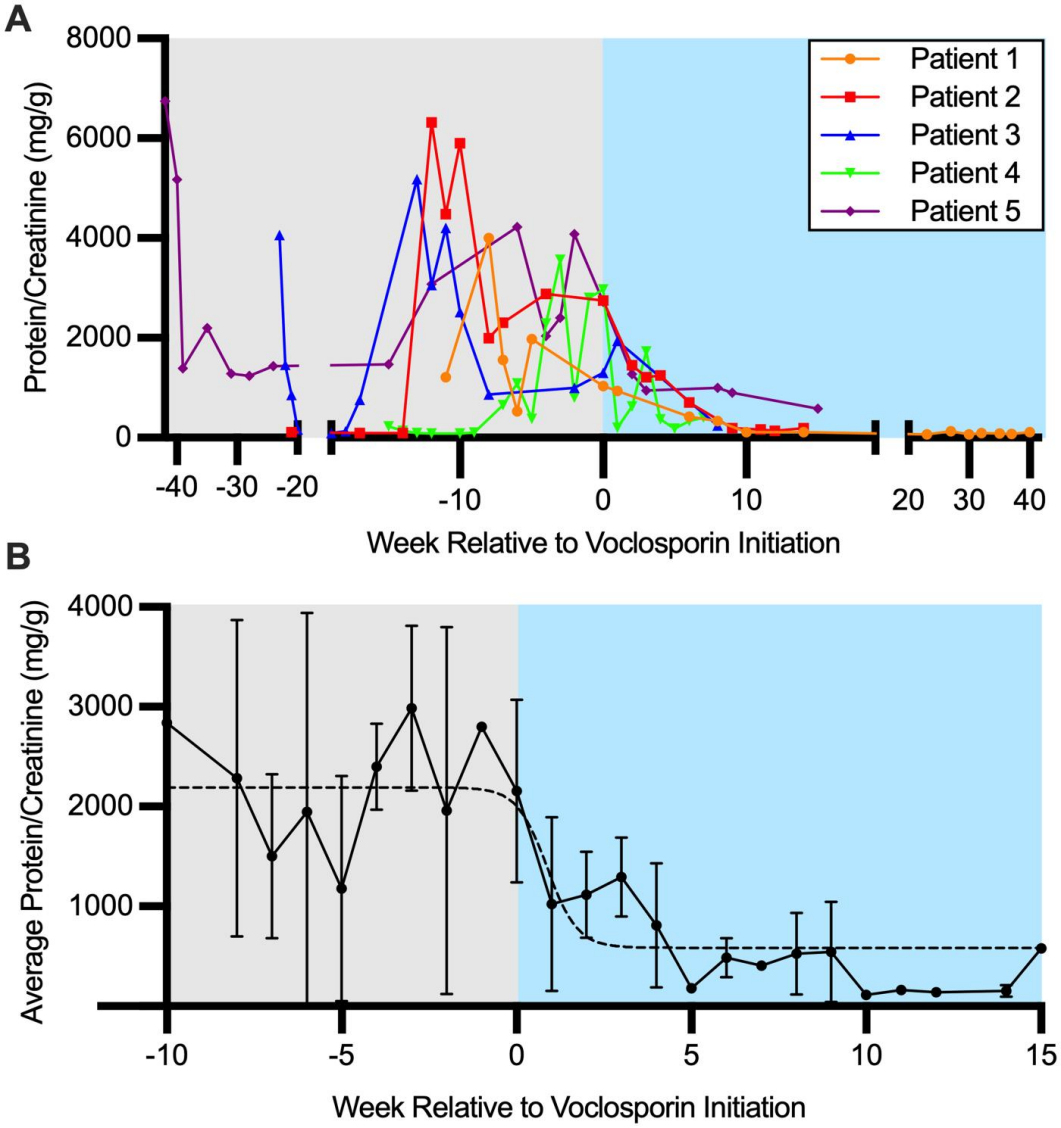
Protein:creatinine ratio is reduced after initiating voclosporin in paediatric lupus nephritis. (A) Urine protein:creatinine measure in mg/g for each patient by week relative to initiation of voclosporin. (B) Average of the urine protein:creatinine measure in mg/g of the five patients by week relative to initiation of voclosporin. Bars representing standard deviation, dashed line representing sigmoidal regression curve

**Patient 1** was diagnosed with SLE at 15 years old, initially presenting with nephrotic-range proteinuria and biopsy-proven class IV LN. After initial remission with MMF, HCQ and steroids, the patient experienced a nephritis flare with hypertension and class V nephritis on repeat biopsy. The patient was started on voclosporin (23.7 mg BID), resulting in significant improvement in proteinuria and sustained renal remission (Fig. 1A). No treatment side effects were noted.

**Patient 2** was diagnosed with SLE at the age of 10. She underwent a biopsy 1 year later after the development of proteinuria, which showed class V LN with minimal activity or chronicity. She was treated with MMF and later initiated on voclosporin (15.8 mg BID). After 14 months of good renal response (Fig. 1A), voclosporin was discontinued due to persistent hypertension, and she transitioned to belimumab. Blood pressure improved; however, prednisone was initiated and discontinued around the same time as voclosporin, making it unclear which medication contributed to hypertension.

**Patient 3** was initially diagnosed with IgA vasculitis that evolved into biopsy-proven IgA/lupus class V nephritis. He also developed additional features of SLE (Supplementary Table S1). Despite multiple treatments including MMF, tacrolimus and IV cyclophosphamide, he had persistent proteinuria and required chronic steroids. After 3 years, voclosporin was initiated, resulting in a substantial reduction in proteinuria, steroid discontinuation and sustained remission (Fig. 1A).

**Patient 4** was diagnosed with SLE and class V nephritis at age 14. She had persistent oedema and proteinuria despite treatment with MMF, HCQ and belimumab. Voclosporin was added due to persistent renal involvement. She experienced rapid improvement in proteinuria (Fig. 1A).

**Patient 5** was diagnosed at age 10 with class IV LN. Her treatment course included cyclophosphamide, rituximab and MMF. Voclosporin was initiated in the context of ongoing LN activity, which resulted in rapid resolution of proteinuria, indicating a successful renal response. After over a year of remission, voclosporin was discontinued (Fig. 1A).

## Conclusion

We report the use of voclosporin in five patients with paediatric LN of varying classes (three with class V, and two with class IV). Our study includes all patients treated with voclosporin at our centre. All five patients received voclosporin due to ongoing disease activity as measured by proteinuria while on standard of care. All patients had a favourable renal response to voclosporin, marked by reduction and/or resolution of proteinuria. Overall, voclosporin was well tolerated and effective for steroid-sparing, remission induction and maintenance, even in refractory or long-standing cases. A limitation of this study is the small sample size and observational design, as well as the use of each patient as an internal control pre- and post-voclosporin while on standard of care therapies. Despite the limitations, our data suggest that voclosporin may be of benefit for the treatment of paediatric LN.

## Supplementary Material

keag125_Supplementary_Data

## Data Availability

The data underlying this article are available in the article and in its online Supplementary material.
