# Supplementary material for: Single-center experience of voclosporin use for pediatric lupus nephritis: a case series
Source: Rheumatology (Oxford). 2026 Mar 15;65(3):keag125. doi: 10.1093/rheumatology/keag125 (PMC13016877; doi:10.1093/rheumatology/keag125)
Supplement: keag125_Supplementary_Data [file keag125_supplementary_data.docx]

*Supplementary material*

**Table S1- Patients characteristics**

| Pt | Age at Dx (yrs) | Sex | Presenting Symptoms | LN Class | Prior Treatments | Time from Dx to voc (mo) | Baseline Pr/Cr Ratio |
| --- | --- | --- | --- | --- | --- | --- | --- |
| 1 | 15 | F | Fatigue, diarrhea, abd pain, petechiae, malar rash, nephrotic range proteinuria, pancytopenia | IV/V* | IVMP, HCQ, MMF, Ritux, IVIG | 11 | 1031 |
| 2 | 10 | F | Fever, weight loss, lymphadenopathy, cytopenia, pericarditis, arthritis | V | IVMP,HCQ, AZA, Ritux, MMF, Myfortic | 21 | 2750 |
| 3 | 10 | M | Purpura, abd pain, arthritis, AIHA, nephrotic range proteinuria | IV | IVMP, HCQ, MMF, Tacrolimus, Belimumab, CYC | 23 | 1298 |
| 4 | 14 | F | rash, arthritis, Raynaud's | V | IVMP, HCQ, MMF, Belimumab | 18 | 2969 |
| 5 | 10 | F | Fatigue, arthritis, rash | IV | IVMP, HCQ CYC, Ritux, MMF | 114 | 2725 |

*initial diagnosed as class IV and later class V

Voc: voclosporin, Abd: abdomen, IVMP: intravenous methylprednisolone, HCQ: hydroxychloroquine, MMF: mycophenolate mofetil, Ritux: Rituximab, IVIG: intravenous immunoglobulin, AZA: azathioprine, CYC: cyclophosphamide.


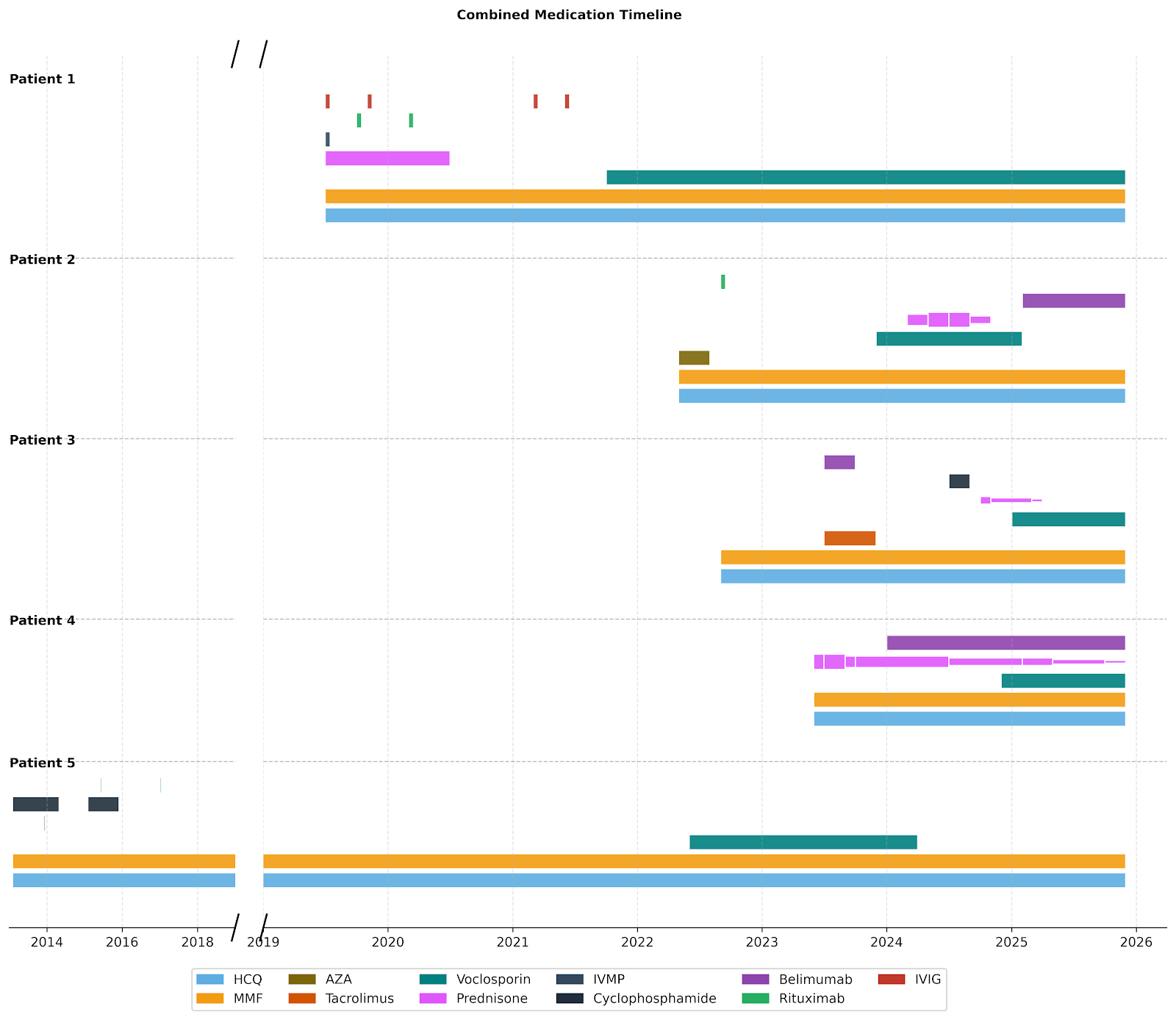


**Supplementary Figure S1. Medication timelines for pediatric lupus nephritis patients treated with voclosporin.** Each horizontal section represents the medication timeline for an individual patient, ordered from top to bottom (Patient 1–5). The timeline is divided into a condensed historical view (2013–2019) and an expanded recent view (2019–Present). Voclosporin is shown in teal, mycophenolate mofetil (MMF) in orange, hydroxychloroquine (HCQ) in light blue, belimumab in purple, rituximab in green, and IVIG in red. Oral prednisone is displayed in pink as a continuous bar, with line thickness proportional to the prescribed dose over time, illustrating tapering or escalation. “Present” indicates ongoing therapy at the time of last follow-up. This visualization highlights the timing and overlap of concomitant immunosuppressive therapies and corticosteroid exposure in relation to voclosporin use.
